# Virtual opioid poisoning education and naloxone distribution programs: A scoping review

**DOI:** 10.1371/journal.pdig.0000412

**Published:** 2024-06-07

**Authors:** Bruna dos Santos, Rifat Farzan Nipun, Anna Maria Subic, Alexandra Kubica, Nick Rondinelli, Don Marentette, Joanna Muise, Kevin Paes, Meghan Riley, Samiya Bhuiya, Jeannene Crosby, Keely McBride, Joe Salter, Aaron M. Orkin

**Affiliations:** 1 Dalla Lana School of Public Health, University of Toronto, Toronto, Canada; 2 Heart to Heart First Aid CPR Services Inc., Toronto, Canada; 3 Canadian Red Cross Opioid Harm Reduction Advisory Council, Canada; 4 The Canadian Red Cross Society, Canada; 5 Department of Family and Community Medicine, University of Toronto, Toronto, Canada; 6 Li Ka Shing Knowledge Institute of Unity Health Toronto, Toronto, Canada; 7 Department of Emergency Medicine, Unity Health Toronto, Toronto, Canada; Iran University of Medical Sciences, IRAN (ISLAMIC REPUBLIC OF)

## Abstract

The global opioid poisoning crisis is a complex issue with far-reaching public health implications. Opioid Poisoning Education and Naloxone Distribution (OPEND) programs aim to reduce stigma and promote harm reduction strategies, enhancing participants’ ability to apply life-saving interventions, including naloxone administration and cardiopulmonary resuscitation (CPR) to opioid poisoning. While virtual OPEND programs have shown promise in improving knowledge about opioid poisoning response, their implementation and evaluation have been limited. The COVID-19 pandemic has sparked renewed interest in virtual health services, including OPEND programs. Our study reviews the literature on fully virtual OPEND programs worldwide. We analyzed 7,722 articles, 30 of which met our inclusion criteria. We extracted and synthesized information about the interventions’ type, content, duration, the scales used, and key findings. Our search shows a diversity of interventions being implemented, with different study designs, duration, outcomes, scales, and different time points for measurement, all of which hinder a meaningful analysis of interventions’ effectiveness. Despite this, virtual OPEND programs appear effective in increasing knowledge, confidence, and preparedness to respond to opioid poisoning while improving stigma regarding people who use opioids. This effect appears to be true in a wide variety of populations but is significantly relevant when focused on laypersons. Despite increasing efforts, access remains an issue, with most interventions addressing White people in urban areas. Our findings offer valuable insights for the design, implementation, and evaluation of future virtual OPEND programs.

## Author summary

The opioid crisis is a global health issue that affects people of all backgrounds, leading to high death rates. Educational programs on opioid poisoning are emerging as life-saving and cost-effective solutions. This review focuses on these programs delivered virtually, removing the need for face-to-face interaction. We’ve gathered and summarized evidence on the outcomes of these programs, which may include naloxone distribution. Our findings provide valuable insights for planning, implementing, and evaluating such programs. We also point out areas where knowledge is lacking, setting the stage for future research.

## Introduction

The opioid poisoning crisis is a global health challenge with high fatality rates [1–3]. The highest rates of opioid poisoning fatalities are seen in the United States, followed by Estonia, Canada, and Lithuania [2]. Opioid-related fatalities have been exacerbated by the increasingly toxic and unregulated drug supply market, contaminated by fentanyl and other substances such as benzodiazepines [2,4,5]. Beyond fatal opioid poisonings, the majority of opioid poisonings are non-fatal and contribute to the social, health, and economic costs of the global opioid poisoning epidemic [6–8]. Opioid-related harms can affect people in all communities, ages, and socioeconomic groups, including family members, friends, healthcare professionals, and community members of people with lived or living experience (PWLLE) of opioid use [9,10].

Opioid Poisoning Education and Naloxone Distribution (OPEND) programs are strategies to train and equip people to provide life-saving interventions, including naloxone administration, to potential opioid poisoning events [11–14]. While existing evidence demonstrates that OPEND can save lives, improve knowledge about opioid poisoning response, improve attitudes, and reduce stigma toward people who use substances, a significant research gap exists [11–16]. Specifically, OPEND programs have predominantly been focused on in-person interventions, which may inequitably inhibit access for those who are unable or unwilling to access in-person services, including rural and remote populations [13,14,17,18]. The COVID-19 pandemic prompted a shift away from in-person offerings, increasing interest in the implementation and evaluation of virtual OPEND programming [19,20]. However, there are no current knowledge syntheses on virtual OPEND programs.

Therefore, this scoping review reports on the existing literature on opioid poisoning education conducted entirely without in-person interaction. In compiling a cohesive overview of existing virtual OPEND programs and their outcomes reported in the literature, our goal is to support the development and evaluation of future programs.

## Methods

### Objective and review question

We synthesized the literature on virtual OPEND programming. We structured our scoping review question based on the Participants, Concept, Context framework for scoping reviews [21], as follows:

- Participants: People at risk of opioid poisoning, likely to witness opioid poisoning, or otherwise interested in OPEND program participation.
- Concept: Any opioid poisoning education program conducted entirely at a distance, without in-person interaction between participants and staff.
- Context: Worldwide.

### Language and terminology

Although ‘overdose’ is a more common term, we use the term ‘poisoning’ throughout this paper. The word ‘overdose’ suggests that the primary cause of the health emergency is an incorrect dose, which most of the time is not necessarily the case and can place stigmatizing blame on people who experience opioid poisoning. Therefore, we suggest using the term ‘poisoning’, which can shift the attention to the broader social context leading to these emergencies, such as the unregulated drug supply. We also prefer and refer here to the term ‘opioid poisoning education and naloxone distribution’ (OPEND) over ‘take-home naloxone’ because the latter does not fully acknowledge the circumstances of the many unhoused people and the educational and non-pharmacological elements that are essential to these programs.

### Approach and protocol

The design and methods for this review are reported following the Preferred Reporting Items for Systematic Reviews and Meta-Analyses Extension for Scoping Reviews (PRISMA-ScR) [22]. Further guidance was gained from an adapted version of Arksey and O’Malley’s methodological framework on scoping reviews and the Joanna Briggs Institute methodological guidance for conducting scoping reviews [23,24]. A preliminary search of MEDLINE and the Cochrane Database of Systematic Reviews was conducted, and no current or ongoing literature reviews on the topic were identified. We developed a scoping review protocol and registered it on Open Science Framework [https://doi.org/10.17605/OSF.IO/EW9RQ] [25].

### Search strategy

An initial search was undertaken on MEDLINE to identify papers on the topic using terms such as ‘virtual’, ‘education’, ‘opioids’, and related terminology [21]. The text words contained in the titles and abstracts of relevant papers and index terms were used to develop a full search strategy for OVID MEDLINE, OVID Embase, EBSCO CINAHL, OVID PsycINFO, The Cochrane Library, SCOPUS, and ERIC (S2 Table). A University of Toronto health science librarian provided expertise in optimizing the search strategy. The search strategy, including all identified keywords and index terms, was adapted for each included database. Searches were conducted on June 1, 2023, except for the grey literature search, which was conducted on July 27, 2023. The grey literature search included the Canadian Agency for Drugs and Technologies in Health (CADTH) Grey Matters website, the System for Information on Grey Literature in Europe (OpenGrey), and TRIP Pro. Additionally, we screened the references of included papers to identify additional ones (reference tracking), but no other papers were identified. All search strategies are documented in S2 Table.

### Source of evidence selection

**Fig 1:**
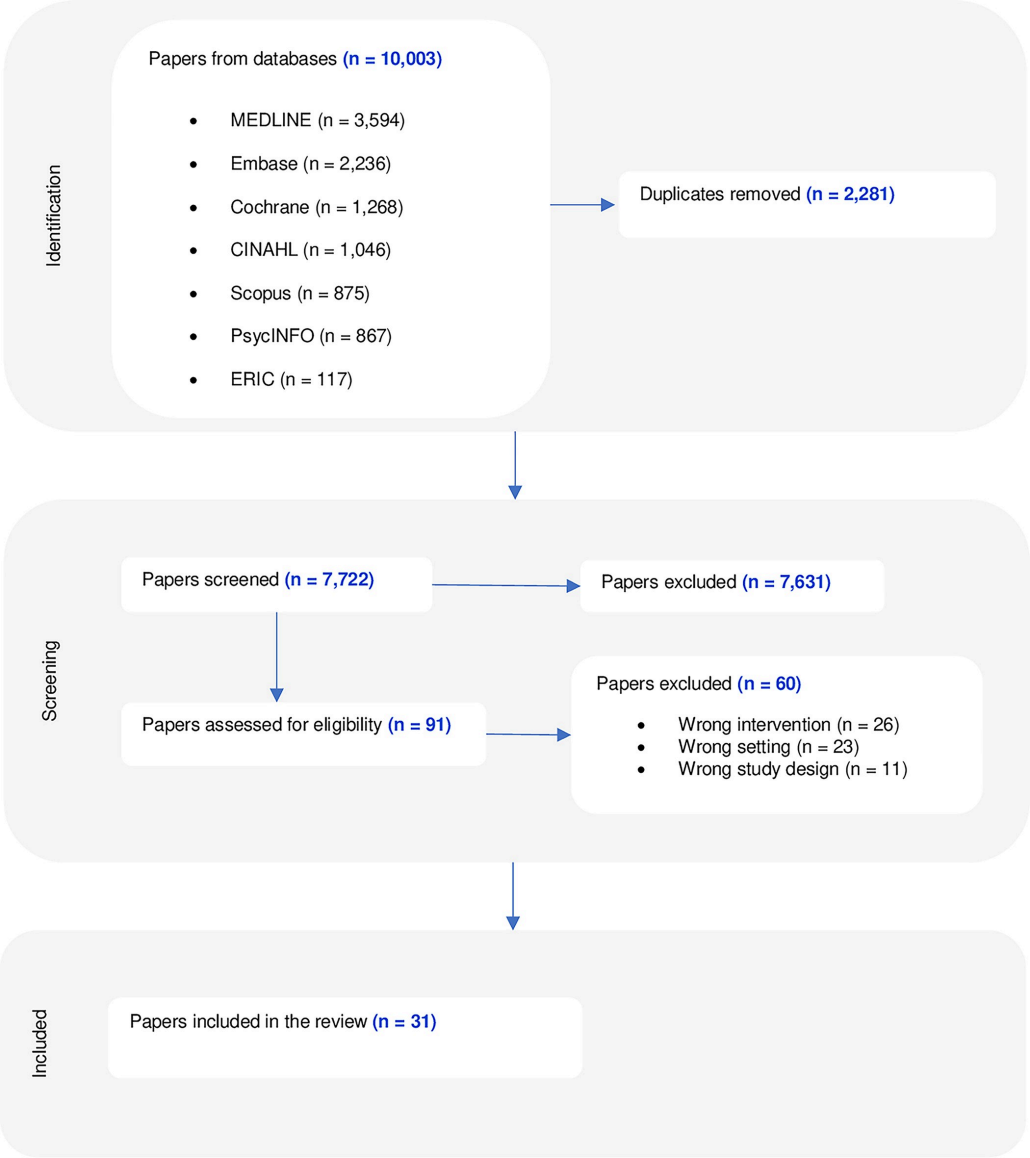
PRISMA flowchart. This figure depicts papers that were included in the scoping review at each stage of the review process, according to PRISMA guidelines.

Following deduplication in Covidence, titles and abstracts were screened independently by four reviewers (BDS, RFN, AK, and AMS) based on the inclusion criteria. The full-text review of selected studies was undertaken by BDS and RFN. Reasons for exclusion during the full-text review were recorded following PRISMA guidelines (Fig 1) [22]. Disagreements between the reviewers at any stage of the selection process were resolved through discussion until a consensus was reached.

### Data extraction

Data were extracted in Covidence from included articles by two independent reviewers (BDS and RFN) using a data extraction tool modified from the JBI Manual for Evidence Synthesis [26]. The data extraction tool was first piloted with five papers per reviewer and modified as necessary. The data extracted included publication details (year of publication, country, and type of study), information about the target population (demographics such as age, gender, and race/ethnicity), intervention components (e.g. videos, telephone calls), topics addressed by the intervention, key findings, and other details relevant to the review question. All variables extracted are provided (S3 Table*)*. Disagreements between the reviewers were resolved through discussion until a consensus was reached.

### Data synthesis

The extracted data are synthesized and summarized in narrative and tabular format. In conducting our analysis, we categorized training into major topic areas, including prevention, response, laws and law enforcement, and developed subtopics to identify the most reported topics addressed in opioid poisoning and naloxone administration training. This analysis was conducted by examining the content of training materials reported in the relevant publication, its citations, or when possible, through a direct review of original educational resources.

## Results

After deduplication, 7,722 out of the initial 10,003 papers were identified for further review. After title and abstract screening, 7,631 papers were excluded, and 91 were reviewed in full text (Fig 1). A full-text review elicited 31 papers for inclusion within this scoping review. Sisson et al., 2023 conducted a single study that was reported in two papers and was, therefore, reported here as a single study [27,28].

The characteristics of the included papers, such as type of study, number and type of participants, sex or gender, and age, are specified in Table 1. Of the papers included in our analysis, 29 (97%) were conducted in the United States of America and one in Australia [29].

**Table 1:** Characteristics of included papers.

Study	Intervention and Participants	Sex or Gender^a^	Age (years)	Duration (minutes)	Scales	Findings
**Adams, 2020[30],** **Pre-post**	Asynchronous video + quiz: 54 law enforcement officers	Male 54.2%	40+ 58.3%	15 to 25	Self-developed	• Increased knowledge about opioids and OUD^b^ maintained for 65% of participants at the 6-month follow-up. • Increased confidence in working with people who use opioids. • Improved attitudes and decreased stigma around OUD^b^. • Participants found the training easy and interesting and would share with others.
**Beiting, 2022[31],** **Pre-post**	Videoconference + mail-delivered naloxone: 51 health professionals	Female 92%	35–64 79%	Not reported	Self-developed	• Increased knowledge regarding the opioid poisoning epidemic. • Increased confidence in recognizing opioid poisoning and administering naloxone. • More participants would prefer in-person training but were still comfortable with a virtual format.
**Bergeria, 2019[32],** **Pre-post**	Asynchronous slide presentation: 61 people with living or lived experience of opioid use Asynchronous slide presentation + quiz: 58 people with living or lived experience of opioid use	Presentation: Male 57% Presentation + quiz: Male 56%	Presentation: M = 34,7 (SD = 11.4) Presentation + quiz: M = 35 (SD = 10.8)	Presentation: M = 27.4 (SD = 15.8) Presentation + quiz: M = 36.4 (SD = 22.3)	BOOK^c^	• Increased knowledge about opioid poisoning immediately after the training, with a small decrease at the 30-day follow-up. • Participants with acute pain had lower knowledge scores than those with chronic pain and illicit opioid use. • Decreased some opioid poisoning risk behaviours. • No significant differences in intervention acceptability between groups. • More participants dropped out of the presentation + quiz group.
**Berland, 2019[33],** **Pre-post**	Asynchronous course with 4 modules: 127 first-year medical students In-person: 130 first-year medical students	N/A	N/A	Not reported	OOKS^d^ (adapted) OOAS^e^ (adapted) MCRS^f^ (adapted)	• Knowledge and preparedness increased in both groups. • No significant changes in attitude scores in either group. • Participants from both groups were satisfied with the training. • There were no meaningful differences between groups.
**Castillo, 2022[34],** **Pre-post**	Asynchronous slide presentation: 7 nurse practitioners	N/A	N/A	20	N/A	• Increased confidence in identifying patients who may be at risk of opioid poisoning and discussing naloxone as a harm reduction strategy. • Increased motivation and intent to prescribe naloxone to patients taking long-term opioids. • The highest perceived barrier to prescribing naloxone was time constraints. • Perceived barriers did not have an impact on willingness to prescribe naloxone. • Participants generally had positive attitudes about naloxone. • Participants rated the intervention at 5.0 out of 5.0.
**Cerles, 2021[35],** **Pre-post.** **Available at rb.gy/sqiyo**	Asynchronous videos: 9 first responders for the pilot and 18 for the intervention	Pilot: Male 78% Female 22% Intervention: Male 61% Female 39%	Pilot: 25–44 56% 45–64 44% Intervention: 25–44 72% 45–64 17% 18–24 6% 65+ 6%	20	N/A	• Most participants had previous workplace opioid exposure training. • Most participants were familiar with virtual learning. • Significant increase in knowledge, not maintained at the 3-month follow-up. • Increased participants’ confidence in carrying out their duties. • Videos got an average rating of 4.9 out of 5.
**Eukel, 2020[36],** **Pre-post.** **Available at oneprogram.org**.	Asynchronous course with 5 modules: 187 pharmacists	Female 78.5% Male 21.5%	N/A	180	Not reported	• Increased knowledge. • Improved behaviours, maintained at the 12-month follow-up. • The overall training rating was 2.7 out of 3. • Most participants recommended the program. • Naloxone benefits were explained to most patients at risk for opioid poisoning.
**Farrugia, 2022[29], Descriptive.** **Available at overdoselifesavers.org**	Website: general population, n not reported	Female 60.7% Male 35.2% Non-binary 1.9% Other 3.9%	30+ 90.3%	Not reported	N/A	• More than half of respondents indicated learning more about naloxone and opioid poisoning. • Most respondents would recommend the website. • Most participants visited the website for professional reasons. • The most accessed pages were ‘Resources and information’, ‘Signs of poisoning and how to respond’, ‘Team and thanks’, and ‘Personal stories’, with the latter reported as the most helpful section. • Addressing stigma and increasing awareness of naloxone were two primary themes recognized from participants’ comments.
**French, 2021[37], Descriptive.** **Available at nextdistro.org**	Asynchronous video + mail-delivered naloxone: 422 from the general population	Cisgender women 54%	M = 33.3 (SD = 11.8)	Not reported	N/A	• Most participants had proximity to opioid poisoning. • The most frequently reported barriers to accessing naloxone were COVID-19-related, lack of knowledge on how to access, cost, transportation, and stigma. • Most common ways individuals learned about the program were through social media and word of mouth.
**Galiher & Huffman, 2022[38],** **Pre-post.** **Available at rb.gy/lwrjs**	Asynchronous video: 219 from the general population	Male 28.8%, Female 71.2%	25–34 31.5% 51–69 26% 35–50 25.6% 18–24 11.4% 70+ 5.5%	6.5	OOAS^e^ (adapted)	• Most participants or their family/close friends had personal experience with substance use. • Most participants did not have previous naloxone training. • Improved self-perception of participants’ ability to manage opioid poisoning. • Significantly improved attitudes toward opioid poisoning. • Participants with lower education levels and/or personal experience with opioid use had higher improvement.
**Giordano, 2020[39],** **Pre-post**	Hybrid simulation: 31 nursing students Asynchronous virtual reality video: 19 nursing students	Hybrid simulation: Female 93.5% Virtual Reality: Female 94.7%	Hybrid simulation: M = 21.06 (SD = 0.44) Virtual Reality: M = 21.06 (SD = 0.42)	Virtual reality: 20	OOKS^d^ OOAS^e^	• None of the participants had experience administering or carrying naloxone before the training. • 12% of participants reported carrying naloxone in a 3-week follow-up, and one administered it. • There were no significant changes in knowledge in both groups. • There was an overall significant decrease in attitude scores. • No difference between groups regarding knowledge and attitudes towards opioid poisoning response.
**Giordano, 2023[40],** **Pre-post.** **Available at virtualinnovation.org**	Asynchronous virtual reality video: 11 nursing students Videoconference: 6 nursing students	Virtual reality: Female 90.9% Other 9.1% Videoconf.: Male 16.7% Female 83.3%	Virtual reality: M = 26.7 (SD = 2.0) Videoconf. M = 25.9 (SD = 4.1)	Virtual reality: 9 Videoconf.: 90	OOKS^d^ OOAS^e^	• Only one participant reported ever administering or carrying naloxone before the training. • VR participants significantly improved their attitudes. • Videoconference participants significantly improved their attitudes and knowledge of poisoning response. • No significant differences between modalities.
**Goss, 2021[41],** **Pre-post**	Asynchronous video + videoconference: 62 first-year medical students In-person: 97 first-year medical students	In-person (2018): M:F 0.75 + 1 prefer not to say In-person (2019): M:F 0.81 + 1 prefer not to say Video (2020): M:F 1.38	In-person (2018): M = 23.3 In-person (2019): M = 23.7 Video (2020): M = 24.5	Video: 50 Videoconf.: 30	OOKS^d^ OOAS^e^ (adapted)	• Most participants did not have previous training in naloxone administration. • There was a significant difference in one knowledge question between groups, with the in-person group scoring higher. • Overall, both groups improved knowledge, attitudes and preparedness toward opioid use and poisoning. • Both groups decreased stigma toward people with OUD^b^.
**Hohmann, 2022[42],** **RCT**	Videoconference: 32 pharmacists No intervention (control): 32 pharmacists	Control: Male 19.4% Female 80.6% Intervention: Male 19.4% Female 77.4%	Control: M = 40.4 (SD = 10.3) Intervention: M = 43.7 (SD = 9.1)	Videoconf.: 90	OOKS^d^ (adapted) OOAS^e^ (adapted)	• Baseline knowledge about the naloxone auto-injector and the nasal spray was higher than for the intramuscular injection and prefilled syringe. • No difference in knowledge about naloxone, perceived barriers to implementation of naloxone, and attitudes towards naloxone, illicit opioid use or prescribed opioid misuse. • 66% of participants felt comfortable providing naloxone to people who use illicit opioids compared to 91% who feel comfortable providing it to people who use prescribed opioids. • Improved confidence in naloxone implementation and logistics. • Increased intention to provide naloxone. • Increased naloxone prescriptions only in the intervention group. • Knowledge, intention, and attitude scores were not associated with the number of naloxone dispensed.
**Hughes, 2022[43], Descriptive**	Telephone + mail-delivered naloxone: 90 PWLLE	N/A	N/A	Not reported	N/A	• From May to June 2020, 165 of the potential patients were reached (33%). • Of the ones reached, 55% accepted to be trained. • Of the ones trained, 93% agreed to receive a naloxone kit.
**Huhn, 2018[44],** **Pre-post**	Asynchronous slide presentation: 97 people currently prescribed an opioid Asynchronous presentation + quiz: 100 people currently prescribed an opioid	Presentation: Female 39.2% Presentation + quiz: Female 51%	Presentation: M = 32.7 (SD = 11.1) Presentation + quiz: M = 33.7 (SD = 11.6)	Presentation: M = 21.5 (SD = 12.3) Presentation + quiz: M = 34.7 (SD = 14.0)	BOOK^c^	• 19.5% of participants reported having gone through opioid poisoning in the past. • Most participants knew about naloxone, but only a few had training or a prescription for it. • Increased knowledge in both groups. • High acceptability for the intervention in both groups. • Participants in the Presentation group were more likely to complete the intervention. • 29.3% of participants in the Presentation + quiz group reported that the intervention took too long.
**Jensen, 2019[45], Descriptive**	In-person: 313 veterans Videoconference: 84 veterans	In-person: Female 5.8 Male 94.2% Videoconf.: Female 6% Male 94%	In-person: 60+ 55.3% 20–59 44.7% Videoconf. 60+ 53.6% 20–59 46.4%	60	N/A	• 21.2% of naloxone was prescribed by the videoconference lead pharmacist during the first 6 months of the service. • Of these prescriptions, 78% of patients attended online sessions. • Fewer people with substance use disorder were reached by the videoconference group.
**Kim, 2016[46], Non-randomized CT**	Asynchronous course: 12 medical residents Reading-only: 11 medical residents	Reading-only: Female 45% Intervention: Female 33%	N/A	Intervention: Mdn = 22.5	Self-developed	• Increased knowledge for both groups, with course participants scoring better than the control group. • No statistical difference between the groups in the global assessment.
**Mathias, 2023[47], Descriptive**	Videoconference: 1,982 from the general population	Women 57.8% Unknown 26.3% Men 15.5% Other 0.4%	Unknown 26.5% 25–34 18.2% 35–44 17.1% 45–54 15.7% 55–64 12.2% 25–7.3% 64+ 3%	120	N/A	• Average of 24 attendees per session. • 60% increase in reach and 110% in trained participants compared to before COVID-19. • Reached urban population centres, rural areas, and substantial racial/ethnic diversity.
**Millikan, 2021[48],** **Pre-post**	Videoconference: 8 adolescents	Female 50% Male 25% Unknown 25%	11–17 M = 14.3	45	Not reported	• No participants reported misusing prescribed opioids in the past year, but two reported knowing someone who has. • Increased knowledge and awareness about opioids, opioid misuse, opioid poisoning risks, and how to recognize the signs of opioid poisoning immediately after training. • 100% of participants stated they were less likely to misuse prescribed opioids or heroin. • 87.5% of participants reported they were more likely to talk to someone if they were concerned about themselves or others.
**Moses, 2021[49],** **Pre-post**	In-person: 124 first-year medical students Videoconference: 238 first-year medical students	In-person: Female 50% Online: Female 49.6%	In-person: M = 23.4 (SD = 2.2) Online: M = 23.3 (SD = 2.5)	60	OOKS^d^ OOAS^e^ MCRS^f^ NaRRC-B^g^	• Increased knowledge, with greater improvement in the in-person group. • Improved attitudes toward naloxone use and distribution in both groups. • 97.2% of participants from both groups enjoyed the training and 99.4% believed future cohorts should receive similar training. • An in-person component was still desired by some students.
**Roe & Banta-Green, 2016[50], Descriptive.** **Available at stopoverdose.org**	Website: 422 from the general population	Female 65%	35+ 57%	Not reported	Self-developed	• Most respondents did not have previous training. • Most participants correctly identified basic signs of opioid poisoning and appropriate responses. • No differences in knowledge scores between respondents indicating professional or personal interest. • Respondents with personal interest were more likely to report being very likely to obtain naloxone. • Respondents had a poor understanding of the local Good Samaritan law.
**Rothbauer, 2022[51], Descriptive**	Telephone + mail-delivered naloxone: 160 veterans with specified opioid prescription risk factors	Male 90%	M = 65	M = 4.85 Mdn = 5	N/A	• 73.8% of potential participants were reached on the first or second attempt. • Of the ones reached, 78% accepted the naloxone kit. • The most common reason to refuse naloxone was feeling it was unnecessary. • Pharmacy students conducting the calls reported satisfaction with the program and wanted to continue.
**Simmons, 2016[52],** **Pre-post.** **Available at getnaloxonenow.org**	Asynchronous course: 387 professional first responders	N/A	N/A	45	OOKS^d^ (adapted) OOAS^e^ (adapted)	• 88.1% of participants felt they had the knowledge and skills to intervene in opioid poisoning after the training. • Most trainees were satisfied or very satisfied with the training content, format, and mode of delivery. • Increased confidence and motivation to administer naloxone. • Most participants would be willing to be retrained 6–12 months after the initial training. • After the training, participants administered naloxone in 88 emergencies, with a 78.4% success rate.
**Simmons, 2018[53],** **Pre-post.** **Available at getnaloxonenow.org**	Asynchronous course: 311 from general population	N/A	N/A	20	OOKS^d^ (adapted) OOAS^e^ (adapted)	• Most respondents reported that they participated in training for professional reasons. • More than 80% of participants were highly satisfied with the training. • 89% of participants felt they had the knowledge and 87.8% the confidence to intervene in opioid poisoning after the training. • While 71.7% of the participants expressed interest in obtaining naloxone, 30.2% reported attempting to do so. • Reported barriers to obtaining naloxone included: not being sure how to obtain it, inability to get a prescription, lack of availability, and cost.
**Sisson, 2023a and 2023b[27,28], RCT**	Asynchronous video: 50 people with living or lived experience of opioid use Asynchronous video + mail-delivered naloxone: 48 people with living or lived experience of opioid use	Video-only: Female 30.6% Male 19.8% Video + mail-delivered naloxone: Female 31.5% Male 18%	Video-only: M = 38.5 (SD = 10.5) Video + mail-delivered naloxone: M = 37.4 (SD = 9.1)	Video: 10 Reading: 10	OOKS^d^ (adapted) OOAS^e^ (adapted)	2023a: • 88% of enrolled individuals completed the training and the 1-month follow-up, 86% completed the 2-month follow-up, and 83% completed the 3-month follow-up. • Mean satisfaction score of 94.63% with the training. • 85.4% of participants in the group with naloxone distribution expressed high satisfaction, while 60% in the education-only group were equally content. 2023b: • Significant increase in knowledge of opioid poisoning and naloxone. • 42% of participants in the education-only group obtained a naloxone kit, mainly the nasal spray. • 16 participants reported using their naloxone over the 3-month follow-up period. • No difference in naloxone utilization between groups. • Most kits were used on friends or family members. • All opioid poisoning reversals resulted in survival.
**Waldron, 2022[54],** **Pre-post.** **Available at rb.gy/60j32**	Asynchronous video: 28 school workers	Female 67,9% Male 28.6% Unknown 3.6%	26–62 M = 46.33 (SD = 10.05)	60 to 90	New General Self-Efficacy Scale (adapted)	• Increased knowledge of how to recognize and respond to opioid poisoning. • Increased confidence in recognizing and responding to opioid poisoning. • Almost all participants were highly satisfied with the content and methods.
**Wu, 2021[55], Descriptive**	Telephone + mail-delivered naloxone: 164 patients receiving prescription opioids	Male 92% Female 8%	60–69 39% 70+ 30% 50–59 19% 39–9% 40–49 3%	M = 14	Not reported	• 67% of potential participants were reached, mostly within one attempt. • Of the ones reached, 73% accepted naloxone. • The most common reasons for declining naloxone were no interest or perceived risk of opioid poisoning, no longer using opioids or trying to stop, and living alone. • Participants preferred the nasal formulation.
**Yang, 2021[56], Descriptive.** **Available at nextdistro.org**	Website + mail-delivered naloxone: 3,926 people with living or lived experience, their friends and family	Female 61% Male 34% Gender nonconforming or Non-binary 4% Transgender 1%	26–45 60% 26–22% 46–65 17% 65+ 1%	Not reported	N/A	• Most participants learned about the website through social media. • Substantial volume of requests for naloxone, most of which were fulfilled. • Instances of naloxone use were largely successful in opioid poisoning reversal (95%).
**Yates et al., 2018[57], Descriptive**	Telephone + mail-delivered naloxone: 41 veterans receiving prescription opioids	Male 97.6%	M = 61	Not reported	N/A	• More than half of the participants were at high risk of opioid poisoning. • Only 4.9% of participants had ever received a naloxone kit. • Most participants were successfully contacted within 3 attempts. • Of the ones reached, 63.4% accepted naloxone. • Participants preferred the nasal formulation.

This table shows the characteristics of the papers included in this scoping review.

^a^All papers reported ‘biological sex’, except for Mathias 2023, Farrugia 2022, French 2021, and Yang 2021.

^b^Opioid Use Disorder (OUD).

^c^Brief Opioid Overdose Knowledge (BOOK).

^d^Opioid Overdose Knowledge Scale(OOKS).

^e^Opioid Overdose Attitudes Scale (OOAS).

^f^Medical Condition Regard Scale (MCRS).

^g^Naloxone-Related Risk Compensation Beliefs Scale (NaRRC-B).

Seventeen studies have a quasi-experimental pre-post design (57%); ten are descriptive (33%); two are randomized controlled trials (7%); and one is a non-randomized controlled trial (3%). The publication period extended from 2016 to 2023, peaking notably in 2022 (27%, n = 8).

### Participant demographics

Eight of the included studies (27%) were designed for people with lived or living experience (PWLLE) of opioid use; six (20%) were for anyone interested, including laypersons; and other six (20%) focused on medical, nursing, and pharmacy students. The remaining studies involved health professionals (13%, n = 4), first responders (7%, n = 2), family members or friends of PWLLE (3%, n = 1), school workers (3%, n = 1), and law enforcement officers (3%, n = 1).

Fourteen studies (47%) reported mean participant ages. Participant ages ranged from 11 to 69 years, with the majority falling between 25 and 64 years. There was notable variation in participation based on biological sex, with thirteen studies of 21 (62%) primarily including female participants and nine (43%) male participants. Most studies (70%, n = 21) reported biological sex rather than gender, with 10 of these presenting only male and female options. Four studies (13%) allowed participants to report their gender, while six (20%) did not provide sex or gender information.

Data on race or ethnicity were not reported in thirteen (43%) of the studies. Among the studies reporting race/ethnicity, all of them had White or Caucasian participants as the majority [27,30,31,35,37–40,42,45,47–49,55–58]. Specific racial identities were reported in a few studies, with Beiting et al. (2022) reporting only the percentage of Black individuals, while Yates et al. (2018) and Moses et al. (2021) reported only the percentage of White individuals.

No papers provided information on participants’ socioeconomic status. However, Huhn et al., 2018 reported that the median household income of participants was US$52,500, while Sisson 2023a reported between US$10,000 and US$49,000. Six studies (20%) provided data on participants’ educational level, with 3 having most participants with a high school degree [27,32,58], 2 with the majority having a college degree [38,54], and two having most with at least a graduate degree [36,42]. The geographical area in which participants resided was mentioned in six studies (20%). In four studies, most participants were from urban areas [33,39,40,42], while two studies specifically focused on rural regions [34,54].

### Intervention approaches

Several strategies were employed for conducting virtual OPEND. A summary of intervention approaches is specified in Table 1, including the type of approach, duration, scales used to measure outcomes and key findings. A substantial number of the included studies (87%, n = 26) delivered their intervention over the internet, including videos (n = 9, with 2 virtual reality simulations), videoconferences (n = 6), courses (n = 5), slide presentations (n = 3), and websites (n = 3). Of the internet-based interventions, four additionally provided naloxone by mail. There were four telephone-based interventions (13%) coupled with mail-delivered naloxone.

Among the 22 studies that reported on duration, interventions ranged from 5 minutes to 2 hours, with most lasting approximately 20 minutes (n = 5, 17%). In four studies (13%), participants could complete the intervention in their own time [29,34,34,36,50]. Of these self-directed interventions [29,36,50], one reported the average time taken for participants to complete their training [36], while none reported drop-out rates.

The 30 studies analyzed in this review incorporated a variety of topics into their interventions. The predominant theme was how to respond to, recognize, and prevent opioid poisoning emergencies. Some commonly mentioned steps were naloxone administration and calling 911. Additional topics included history and statistics on opioid poisoning, laws and law enforcement, specifics on naloxone use, and stigma. An overview of the educational components can be seen in Table 2.

**Table 2:** Topics addressed in trainings.

Major Topics	Subtopics	References
**Statistics and History of Opioid Poisoning**		8
**Prevention of Opioid Poisoning**	Opioid poisoning risks	8
	Contamination by fentanyl and/or other substances	2
**Recognizing an Opioid Poisoning**		19
**Responding to an Opioid Poisoning**	PPE and situational awareness	2
	Calling 911	7
	Airway and circulation assessment	1
	Sternal rub	1
	Naloxone administration	22
	Rescue breathing	3
	Recovery positioning	4
	Opioid withdrawal management	3
	Continued monitoring until EMS arrives	3
**Laws and Law Enforcement**	Barriers to calling 911	2
	Good Samaritan Law	5
	Naloxone Access Law	2
**Naloxone Specifics**	Intranasal vs. intramuscular use	1
	Mechanism of action	2
	Access and availability	1
	Storage and expiration	1
**Stigma about Opioid Poisoning**		6

Most studies utilized a pre-post design or post-training questionnaires. 67% (n = 20) assessed participants’ knowledge of opioid poisoning response, followed by 11 (37%) that gathered qualitative feedback and measured satisfaction rates. Ten studies (33%) focused on attitudes, behaviour, and stigma related to opioid poisoning emergencies, and another 10 tracked naloxone kit distribution, prescriptions, and administration during or after the intervention. Other outcomes included confidence in responding to opioid poisoning, comparisons between different modalities, accessibility, barriers to access naloxone, preparedness, familiarity with virtual learning, and opioid use.

Regarding assessment tools, 33% (n = 10) of studies used the Opioid Overdose Knowledge Scale (OOKS) and the Opioid Overdose Attitudes Scale (OOAS). Most studies (n = 7) adapted the scales for their intervention, while 4 (13%) developed new assessment tools. The Brief Opioid Overdose Knowledge Questionnaire (BOOK) and the Medical Conditions Regard Scale (MCRS) were each used by two studies (7%). Other scales used were the New General Self-Efficacy Scale and the Naloxone-Related Risk Compensation Beliefs Scale.

### Key findings

Findings from the included studies are summarized in Table 1.

#### Videos (n = 9)

*Knowledge and confidence*. Most studies reported an increase in knowledge about opioids, opioid use disorder (OUD), and opioid poisoning, as well as an increase in confidence in managing opioid-related situations [27,30,35,41,54,58]. While Adams et al. (2020) showed that the knowledge increase was sustained for most participants at the 6-month follow-up, in Cerles et al. (2021) this increase was not sustained at the 3-month follow-up.

*Attitudes and stigma*. There was an improvement in attitudes and a decrease in stigma around OUD and opioid poisoning in several studies [30,38,40,41]. Giordano et al. (2020), however, reported a significant decrease in attitude scores.

*Training satisfaction*. Participants generally found the training easy and interesting, and were highly satisfied with the content and methods, giving the videos a high satisfaction rating [27,27,30,35,54].

*Previous experience or training*. Many participants had personal experience with substance use or had proximity to opioid poisoning [37,38]. Most were not previously trained in naloxone administration or had personal experience administering or carrying naloxone [38–41].

#### Videoconferences (n = 7)

*Knowledge and confidence*. Most studies reported an increase in knowledge about the opioid epidemic, opioid misuse, opioid poisoning risks, and naloxone [31,40,48,49]. There was also an increase in confidence in recognizing poisoning and administering naloxone [31,42]. However, Hohmann et al. (2022) found no difference in knowledge about naloxone and perceived barriers to its implementation.

*Attitudes*. Some studies reported improved attitudes toward naloxone use and distribution [40,49]. However, Hohmann et al. (2022) found no difference in attitudes toward naloxone, illicit opioid use, or prescribed opioid misuse.

*Training and satisfaction*. Two papers reported that most participants are comfortable with a virtual format but prefer in-person training [31,49].

*Reach and diversity*. Mathias et al. (2023) reported reaching both urban and rural populations, and substantial racial/ethnic diversity. Notably, Jensen et al. (2019) found that fewer people with substance use disorder were reached in the videoconference compared to the in-person group.

#### Courses (n = 5)

*Knowledge*, *confidence and preparedness*. All studies reported an increase in knowledge [33,36,46,52,53]. Additionally, Berland et al. (2019) reported increased preparedness, while Simmons et al. (2016) and (2018) reported increased confidence to intervene in opioid poisoning.

*Attitudes and behaviour*. Eukel et al. (2020) reported improved behaviours at the 12-month follow-up. However, Berland et al. (2019) reported no significant changes in attitude scores.

*Training and satisfaction*. Four studies reported high participant satisfaction rates with the courses [33,36,52,53].

#### Telephone calls (n = 4)

*Reach and acceptance*. All studies reported that a significant portion of potential participants were reached [43,51,55,57]. Most patients were reached within one, two or three attempts [51,55,57].

#### Slide presentations (n = 3)

*Knowledge and confidence*. Two studies reported increased knowledge and one increased confidence about opioid use and poisoning after the intervention [32,34,44]. Bergeria et al. (2019) noted a small decrease in knowledge at the 30-day follow-up.

*Attitudes and stigma*. One study found that the training was effective in decreasing stigma around prescribing naloxone [34]. Another reported fewer opioid poisoning-risk behaviours after the intervention [32].

*Training and satisfaction*. All studies reported high levels of satisfaction or acceptability for their interventions [32,34,44].

#### Websites (n = 3)

*Knowledge*, *confidence and awareness*. One study reported increased knowledge and confidence in discussing opioid poisoning [29], and another showed that most participants, with either a professional or personal interest in the training, correctly identified basic signs of opioid poisoning and appropriate responses [50].

#### Naloxone

Of the interventions that offered naloxone by mail, four were telephone-based [43,51,55,57], two were videos [27,28,37], one was a videoconference [31], and another was a website [56]. Some studies, though not distributing naloxone, reported on naloxone-related outcomes.

*Acceptability*. Acceptability rates for naloxone kits distribution ranged from 73% to 93% [43,51,55,57]. Participants with a personal interest in the training were more likely to report being very likely to obtain naloxone [50]. The most common reason participants did not accept naloxone was because they felt it was unnecessary [51]. Castillo et al. (2022) reported an increase in nurses’ motivation and intent to prescribe naloxone to patients taking long-term opioids. Similarly, Hohmann et al. (2022) found that 66% of participants felt comfortable providing naloxone to people who use illicit opioids compared to 91% who felt comfortable providing it to people with prescribed opioids.

*Barriers*. The most frequently reported barriers to obtaining naloxone were related to the COVID-19 pandemic, lack of knowledge on how to access it, inability to get a prescription, cost, lack of availability, transportation, and stigma [37,53]. Despite perceived barriers not impacting the willingness to prescribe naloxone, the highest perceived barrier for nurses was time constraints [34].

*Naloxone use and outcomes*. Most or all opioid poisoning reversals resulted in survival, and the majority of kits were used on participants’ friends or family members [28,52,56]. Giordano et al. (2020) reported that, though they did not distribute naloxone kits, 12% of participants reported carrying naloxone in a 3-week follow-up, and one administered it. Sisson et al. (2023b) reported that 42% of individuals in the groups without naloxone distribution obtained their kit.

#### Comparison between different modalities

*Comparison groups*. Four studies had a control group involving in-person training [33,41,45,49], and one study had a hybrid group [39]. Other comparison groups included reading-only or course, slide or slide + quiz, and virtual reality video or videoconference.

*Differences between formats*. Three studies with an in-person or hybrid group showed no significant differences in preparedness, attitudes, satisfaction, and stigma scores between formats [33,39,41], and one reported greater knowledge in the in-person group [49]. One study reported a significantly higher number of naloxone prescriptions obtained by participants in the virtual group [45]. For papers with other comparison groups, there were also no significant differences between groups [32,40,46]. Huhn et al. (2018) reported that the presentation-only group was more likely to complete the intervention compared to the group with the presentation coupled with a quiz [44]. Sisson et al. (2023a) reported higher satisfaction in the naloxone distribution group compared to the group without naloxone distribution [58]. Hohmann et al. (2022) showed an increase in the number of naloxone prescriptions obtained by participants only in the intervention group, but there was no difference related to other outcomes [42].

#### Limitations

The included studies reported limitations in their designs. Twenty studies (67%) reported a lack of generalizability of their study results [27,28,30–35,39–42,47–53,55,57]. Another frequent obstacle reported in 12 studies is the small sample size (40%), which ranged from six to 313 participants per group [30–32,34,35,39,40,42,45,48,51,54]. Other limitations include the absence of longitudinal data [31,37,42,44,48,50,55], self-reported bias for questionnaires [27,28,49,52,53], the lack of comparison to an in-person intervention [38,41,44,47], no follow-up on the number of participants who responded to an opioid poisoning post-intervention [37,45,47,55], lack of validity of the tools used [30,34,36], low response rates of 21.2% and 33.7% [36,53], and the lack of randomization [46,47].

## Discussion

This scoping review identified that there is considerable research and innovation underway in the implementation and evaluation of virtual OPEND, particularly since the start of the COVID-19 pandemic. Although this review did not include a critical appraisal, it is crucial to note that the diverse study designs, varying intervention durations, and different measurement time points complicate the analysis of intervention effectiveness.

Despite current challenges in analyzing intervention effectiveness, it is important to highlight the positive outcomes reported. Overall, virtual OPEND appears to be acceptable to participants and effective in increasing knowledge on how to respond to opioid poisoning, as well as improving attitudes and decreasing stigma about people who use opioids. Especially when coupled with naloxone distribution, programs appear to impact the motivation and willingness of people to respond to these emergencies. Notably, some studies demonstrated a high rate of successful resuscitations and decreased poisoning death rates [27,28,52,56]. Therefore, while the diverse study designs present analytical challenges, they do not detract from the potential life-saving impact of virtual OPEND.

Our synthesis indicates that most interventions are primarily targeted at PWLLE of opioid use and potential bystanders, defined as individuals who could potentially witness opioid poisoning. A cross-sectional internet-based survey and a mixed-methods study have demonstrated the unique position of bystanders in reversing opioid poisonings and saving lives when they are equipped with naloxone and the knowledge to administer it effectively [59,60].

The bulk of these studies are based in the USA, with only one study conducted in Australia [29]. However, opioid poisoning is a global concern, which demonstrates a pressing need to implement and evaluate virtual OPEND programs worldwide [2,13]. Most studies had a pre-post design, followed by descriptive studies, RCTs, and one non-randomized controlled trial, and did not include longitudinal data. Therefore, there is a need for more randomized and controlled trials that also assess the attrition of knowledge and the potential need or importance of retraining so more effective interventions can be designed and implemented in the future.

In addition, only 42% of studies utilized validated scales to measure outcomes related to opioid poisoning, with 62% of these studies using a modified version of the validated scales. These validated scales, especially OOKS and OOAS, were originally designed to assess illicit opioid poisoning risk factors and do not include knowledge specifically about naloxone [61,62]. Therefore, there is a critical demand for updated and validated survey instruments to effectively evaluate these interventions [61,62].

Few studies reported sociodemographic variables and focused on the inclusion of historically marginalized populations. Yet, those more likely to be impacted by the opioid poisoning crisis include people living in rural and remote areas, individuals experiencing homelessness, those living in poverty, incarcerated individuals, and Black, Indigenous, and People of Colour [63]. Without sociodemographic information, it is unknown whether sampling and selection biases and issues with representativeness have occurred, limiting the effectiveness of these interventions in addressing health inequities [64].

Moreover, most interventions did not make their training materials publicly available, and some of them did not report the educational content included in the intervention, hindering the potential for public dissemination and research replication. Among the studies that reported the content of their training, stigma reduction and policies on witness protection and naloxone use permission for opioid poisoning reversal were frequently not addressed, which if included, could have bolstered the outcomes [65,66].

This is the first scoping review on fully virtual OPEND interventions, highlighting the outcomes and potential impacts of these programs. Other reviews have explored opioid poisoning interventions and their effectiveness but did not focus on virtual programming. Pellegrino et al., (2021) conducted a scoping review on first aid educational interventions for opioid poisoning published until 2019, showing the potential of these programs with most studies showing improved educational and clinical outcomes, despite the sparsity and lack of quality of the studies reviewed [14]. Razaghizad et al. (2021) conducted an umbrella review on OPEND, demonstrating that there is credible evidence that opioid poisoning education programs improve knowledge and attitudes about opioid poisoning, enable participants to use naloxone safely and effectively, and reduce opioid-related mortality [13]. Additionally, Tas et al. (2022) reviewed technological interventions that prevent, detect, or respond to opioid poisoning emergencies, with promising approaches that were still under development, such as smartphone apps for alerting and recruiting first aid volunteers to act on nearby opioid poisoning events [67].

Our findings suggest the potential effectiveness of virtual OPEND interventions as a lifesaving tool, and can assist researchers and public health practitioners in designing, implementing, and evaluating future OPEND programming. We recommend providing more funding, personnel, and technology to amplify the implementation and evaluation of these programs. We believe coupling first-aid opioid poisoning training with a well-structured naloxone distribution system can notably increase the positive outcomes of this intervention. To address inequities and specific local challenges, we recommend sociodemographic information to be collected and interventions to be tailored by working closely with local communities and governments. It is also crucial to invest in the development or update and validation of relevant evaluation tools that can better assess OPEND’s effectiveness for diverse outcomes and in diverse settings. Additionally, we suggest conducting longitudinal studies to address the long-term outcomes of these interventions, including the duration of knowledge and the necessity for retraining.

## Limitations

Our review has limitations. One such limitation is the lack of extensive and evidence-informed critical appraisal of the studies included. For this reason, it is not possible to indicate the quality of the studies or extract a meaningful conclusion. In the context of diverse study designs, different methodologies, intervention durations, and measurement time points can introduce variability that makes it challenging to compare results across studies. The variability of the studies makes it unclear whether the results synthesized in our review can be extrapolated to other settings, study designs or populations. Despite our best efforts to design a comprehensive search strategy without imposing any limitations, the constant updates and rapid publication of new research on technology-based interventions may result in the unintentional omission of important studies by the time of publication. We also acknowledge that many impactful programs addressing the opioid poisoning crisis are currently available online but did not involve a research design or share publicly available data on outcomes of these interventions and, therefore were not suitable for this review. Additionally, the lack of detailed information about the interventions in many studies may have impacted the accuracy of our synthesis, as we did not reach out to the authors for additional information.

## Conclusion

This review synthesizes the literature on virtual Opioid Poisoning Education and Naloxone Distribution (OPEND) programs, and underscores gaps in program implementation and evaluation. Currently, it is difficult to draw substantial conclusions on OPEND effectiveness due to the variety of study designs and the lack of updated and validated evaluation methods. However, existing evidence points to the potential to increase knowledge, decrease stigma, and ultimately reduce morbidity and mortality due to opioid-related harms, which enhances its potential as a life-saving tool. This life-saving knowledge can have substantial and positive impacts on populations who are not currently being adequately reached by in-person OPEND interventions.

## Supporting information

S1 Table PRISMA checklist. (PDF)

S2 Table Search strategy. (DOCX)

S3 Table Data extraction instrument. (DOCX)
