# Supplementary material for: Virtual opioid poisoning education and naloxone distribution programs: A scoping review
Source: PLOS Digit Health. 2024 Jun 7;3(6):e0000412. doi: 10.1371/journal.pdig.0000412 (PMC11161022; doi:10.1371/journal.pdig.0000412)
Supplement: S2 Table — (DOCX) [file pdig.0000412.s002.docx]

## S2 Table. Search strategy.

| Database | Search Strategy | Results |
| --- | --- | --- |
| OVID Medline | Concept #1 (combine with OR):  computer simulation/ or augmented reality/ or virtual reality/  Computer-Assisted Instruction/  Education, Distance/  internet/ or internet access/ or "internet of things"/ or "internet use"/ or internet-based intervention/ or social media/  software/ or mobile applications/ or software design/ or software validation/  cell phone/ or smartphone/ or text messaging/  Pharmaceutical Services, Online/  Electronic Mail/  telemedicine/ or telenursing/ or telephone/ or videoconferencing/  call centers/  hotlines/  technology/ or educational technology/   (virtual OR remote OR online OR web* OR computer* OR video* OR smartphone OR “text messag*” OR telephone* OR “mobile device” OR phone* OR “electronic mail” OR e-mail OR “mobile app*” OR hotline* OR helpline* OR “call center*” OR e-health OR m-health OR “mobile health” OR telemedic* OR telenursing OR teleconferenc* OR telehealth OR “digital health” OR website OR software OR technolog* OR “augmented reality” OR internet OR “social media”).tw,kf.    Concept #2 (combine with OR):  education/ or curriculum/ or education, nonprofessional/ or education, professional/ or inservice training/ or teaching/  exp Education, Nursing/ or exp Interprofessional Education/ or exp Education, Medical/ or exp Competency-Based Education/ or exp Education, Public Health Professional/ or exp Education, Pharmacy/  health education/ or health promotion/ or patient education as topic/  exp Learning/  problem-based learning/  simulation training/ or patient simulation/   (educat* OR curricul* OR learn* OR training OR course OR teach* simulation OR instruc* OR “skill-building” OR “skills-building" OR lecture OR coach* OR certifi* OR “health promotion”).tw,kf.    Concept #3 (combine with OR):  exp Analgesics, Opioid/ OR exp Opiate Alkaloids/ OR exp Opiate Overdose/ OR exp Naloxone/   (opioid* OR opiate* OR heroin OR fentanyl OR oxycodone OR hydrocodone OR morphine OR codeine OR naloxone OR narcan OR narkan OR talwin).tw,kf.    Concept #4:  (#2 AND #3) OR ((exp Naloxone/ OR (naloxone OR narcan OR narkan OR talwin).tw,kf. AND (distribution OR provision OR dispensing OR delivery).tw,kf.)    Final Search Strategy: #1 AND #4 | 3594 on 01/06/2023 |
| EBSCO CINAHL | Concept 1 (combine with OR):  (MM "Computer Simulation") OR (MM "Augmented Reality")  (MM "Computer Assisted Instruction")  (MM "Education, Non-Traditional") OR (MM "MOOC") OR (MM "Online Education")  (MM "Internet") OR (MM "Internet Access") OR (MM "Internet-Based Intervention") OR (MH "Social Media+") OR (MM "Internet of Things")  (MM "Software") OR (MM "Mobile Applications") OR (MM "Communications Software+")  (MM "Software Design") OR (MM "Virtual Reality")  (MM "Cellular Phone") OR (MM "Text Messaging") OR (MM "Smartphone")  (MM "Online Services") OR (MM "Email") OR (MM "Teleconferencing") OR (MM "Telehealth") OR (MM "Telemedicine") OR (MM "Telenursing") OR (MM "Videoconferencing")  (MM "Telephone Information Services")  (MM "Technology") OR (MM "Educational Technology")   TI ( (virtual OR remote OR online OR web* OR computer* OR video* OR "text messag*" OR telephone* OR "mobile device" OR phone* OR "e-mail" OR "mobile app*" OR hotline OR helpline OR "call center" OR "e-health" OR "m-health" OR "mobile health" OR telemedic* OR telenursing OR telehealth OR teleconferenc* OR "digital health" OR website OR software OR technolog* OR “augmented reality” OR internet OR “social media”) OR AB ( (virtual OR remote OR online OR web* OR computer* OR video* OR "text messag*" OR telephone* OR "mobile device" OR phone* OR "e-mail" OR "mobile app*" OR hotline OR helpline OR "call center" OR "e-health" OR "m-health" OR "mobile health" OR telemedic* OR telenursing OR telehealth OR "digital health" OR website OR software OR technolog* OR “augmented reality” OR internet OR “social media”)    Concept #2 (combine with OR):  (MH "Education+") OR (MM "Curriculum+") OR (MM "Education, Competency-Based") OR (MM "Education, Nonprofessional+") OR (MM "Education, Health Sciences+") OR (MM "Education, Clinical+") OR (MM "Health Education")  (MM "Health Promotion")  (MM "Patient Education")  (MM "Teaching+")  (MM "Learning+")  (MM "Simulations") OR (MM "Patient Simulation")   TI ( (educat* OR curricul* OR learn* OR training OR course OR teach* simulation OR instruc* OR "skill-building" OR "skills-building" OR lecture OR coach* OR certifi* OR "health promotion") ) OR AB ( (educat* OR curricul* OR learn* OR training OR teach* simulation OR instruc* OR "skill-building" OR "skills-building" OR lecture OR coach* OR certifi* OR "health promotion") )    Concept #3 (combine with OR):  (MM "Opioid Epidemic") OR (MM "Analgesics, Opioid+") OR (MM "Naloxone")  (MM "Opiate Overdose")   TI ( (opioid* OR opiate* OR heroin OR fentanyl OR oxycodone OR hydrocodone OR morphine OR codeine OR naloxone OR narcan OR narkan OR talwin) ) OR AB ( (opioid* OR opiate* OR heroin OR fentanyl OR oxycodone OR hydrocodone OR morphine OR codeine OR naloxone OR narcan OR narkan OR talwin) )    Concept #4:  (#2 AND #3) OR ((MM "Naloxone") OR TI ( (naloxone OR narcan OR narkan OR talwin) )) OR (AB ( (opioid* OR opiate* OR heroin OR fentanyl OR oxycodone OR hydrocodone OR morphine OR codeine OR naloxone OR narcan OR narkan OR talwin) )) AND (TI (distribution OR provision OR dispensing OR delivery)) OR (AB (distribution OR provision OR dispensing OR delivery))    Final Search Strategy: #1 AND #4 | 1046 on  01-06-23 |
| SCOPUS | Concept #1 (combine with OR):  TITLE-ABS-KEY (virtual OR remote OR online OR web* OR computer* OR video* OR "text messag*" OR telephone* OR "mobile device" OR phone* OR "e-mail" OR "mobile app*" OR hotline OR helpline OR "call center" OR "e-health" OR "m-health" OR "mobile health" OR telemedic* OR telenursing OR telehealth OR teleconferenc* OR "digital health" OR website OR software OR technolog* OR “augmented reality” OR internet OR “social media”)    Concept #2 (combine with OR):  TITLE-ABS-KEY (educat* OR curricul* OR learn* OR training OR course OR teach* simulation OR instruc* OR "skill-building" OR "skills-building" OR lecture OR coach* OR certifi* OR "health promotion")    Concept #3 (combine with OR):  TITLE-ABS-KEY (opioid* OR opiate* OR heroin OR fentanyl OR oxycodone OR hydrocodone OR morphine OR codeine OR naloxone OR narcan OR narkan OR talwin)    Concept #4:  (#2 AND #3) OR TITLE-ABS-KEY ((naloxone OR narcan OR narkan OR talwin) AND (distribution OR provision OR dispensing OR delivery))    Final Search Strategy: #1 AND #4 | 875 on 01/06/2023 |
| OVID PsycINFO | Concept #1 (combine with OR):  computer simulation/ or augmented reality/ or virtual reality/ or Computer-Assisted Instructions/ or distance education/ or electronic learning/ or internet/ or social media/ or websites/ or Internet Usage/ or digital interventions/ or computer software/ or mobile applications/ or mobile health/ or mobile learning/ or mobile phones/ or smartphones/ or text messaging/ or computer mediated communication/ or telemedicine/ or teleconferencing/ or Telephone Systems/ or videoconferencing/ or video-based interventions/ or hot line services/ or technology/    (virtual OR remote OR online OR web* OR computer* OR video* OR smartphone OR “text messag*” OR telephone* OR “mobile device” OR phone* OR “electronic mail” OR e-mail OR “mobile app*” OR hotline* OR helpline* OR “call center*” OR e-health OR m-health OR “mobile health” OR telemedic* OR telenursing OR teleconferenc* OR telehealth OR “digital health” OR website OR software OR technolog* OR “augmented reality” OR internet OR “social media”).tw    Concept #2 (combine with OR):  education/ or adult education/ or curriculum/ or nontraditional education/ or nursing or education/ or medical education/ or health education/ or drug education/ or public health campaigns/ or health promotion/ or exp Teaching/ or exp Learning/    (educat* OR curricul* OR learn* OR training OR course OR teach* simulation OR instruc* OR “skill-building” OR “skills-building" OR lecture OR coach* OR certifi* OR “health promotion” OR “public health campaigns”).tw    Concept #3 (combine with OR):  exp opioid analgesics/ or exp opiates/ or Exp opioid epidemic/ or exp Naloxone/    (opioid* OR opiate* OR heroin OR fentanyl OR oxycodone OR hydrocodone OR morphine OR codeine OR naloxone OR narcan OR narkan OR talwin).tw    Concept #4:  (#2 AND #3) OR (exp Naloxone/ OR ((naloxone OR narcan OR narkan OR talwin).tw AND (distribution OR provision OR dispensing OR delivery).tw))    Final Search Strategy: #1 AND #4 | 867 on 01/06/2023 |
| OVID EMBASE | Concept #1 (combine with OR):  computer simulation/ or augmented reality/ or virtual reality/  Computer-Assisted Instruction/  Education, Distance/  internet/ or internet access/ or "internet of things"/ or "internet use"/ or internet-based intervention/  social media/  software/ or software design/ or software validation/  mobile applications/ or cell phone/  Smartphone/  text messaging/  Pharmaceutical Services, Online/  Electronic Mail/  telemedicine/ or telenursing/  Telephone/  videoconferencing/  call centers/  hotlines/  technology/ or educational technology/   (virtual OR remote OR online OR web* OR computer* OR video* OR smartphone OR “text messag*” OR telephone* OR “mobile device” OR phone* OR “electronic mail” OR e-mail OR “mobile app*” OR hotline* OR helpline* OR “call center*” OR e-health OR m-health OR “mobile health” OR telemedic* OR telenursing OR teleconferenc* OR telehealth OR “digital health” OR website OR software OR technolog* OR “augmented reality” OR internet OR “social media”):ti,ab,kw    Concept #2 (combine with OR):  education/ or curriculum/ or education, nonprofessional/ or education, professional/ or inservice training/ or teaching/  exp Education, Nursing/ or exp Interprofessional Education/ or exp Education, Medical/ or exp Competency-Based Education/ or exp Education, Public Health Professional/ or exp Education, Pharmacy/  health education/ or health promotion/ or patient education as topic/  exp Learning/  problem-based learning/  simulation training/ or patient simulation/   (educat* OR curricul* OR learn* OR training OR course OR teach* simulation OR instruc* OR “skill-building” OR “skills-building" OR lecture OR coach* OR certifi* OR “health promotion”).tw,kf.    Concept #3 (combine with OR):  exp Analgesics, Opioid/  exp Opiate Alkaloids/  exp Opiate Overdose/  exp Naloxone/   (opioid* OR opiate* OR heroin OR fentanyl OR oxycodone OR hydrocodone OR morphine OR codeine OR naloxone OR narcan OR narkan OR talwin).tw,kf.    Concept #4:  (#2 AND #3) OR ((exp Naloxone/ OR (naloxone OR narcan OR narkan OR talwin).tw,kf. AND (distribution OR provision OR dispensing OR delivery).tw,kf.)    Final Search Strategy: #1 AND #4 | 2236 on 01/06/2023 |
| Cochrane | Concept #1 (combine with OR):  [mh ^"computer simulation"] or [mh ^"augmented reality"] or [mh ^"virtual reality"] or [mh ^"Computer-Assisted Instruction"] or [mh ^"Education, Distance"] or [mh ^internet] or [mh ^"internet access"] or [mh ^"internet of things"] or [mh ^"internet use"] or [mh ^"internet-based intervention"] or [mh ^"social media"] or [mh ^software] or [mh ^"mobile applications"] or [mh ^"software design"] or [mh ^"software validation"] or [mh ^"cell phone"] or [mh ^smartphone] or [mh ^"text messaging"] or [mh ^"Pharmaceutical Services, Online"] or [mh ^"Electronic Mail"] or [mh ^telemedicine] or [mh ^telenursing] or [mh ^telephone] or [mh ^videoconferencing] or [mh ^"call centers"] or [mh ^hotlines] or [mh ^"technology"] or [mh ^"educational technology"]  (virtual OR remote OR online OR web* OR computer* OR video* OR smartphone OR "text messag*" OR telephone* OR "mobile device" OR phone* OR "electronic mail" OR e-mail OR "mobile app*" OR hotline* OR helpline* OR "call center*" OR e-health OR m-health OR "mobile health" OR telemedic* OR telenursing OR teleconferenc* OR telehealth OR "digital health" OR website OR software OR technolog* OR "augmented reality" OR internet OR "social media"):ti,ab,kw    Concept #2 (combine with OR):   [mh ^"education"] or [mh ^"curriculum"] or [mh ^"education, nonprofessional"] or [mh ^"education, professional"] or [mh ^"inservice training"] or [mh ^"teaching"]   [mh "Education, Nursing"] or [mh "Interprofessional Education"] or [mh "Education, Medical"] or [mh "Competency-Based Education"] or [mh "Education, Public Health Professional"] or [mh "Education, Pharmacy"]   [mh ^"health education"] or [mh ^"health promotion"] or [mh ^"patient education as topic"]   [mh "Learning"]   [mh ^"problem-based learning"]   [mh ^"simulation training"] or [mh ^"patient simulation"]  (educat* OR curricul* OR learn* OR training OR course OR teach* simulation OR instruc* OR "skill-building" OR "skills-building" OR lecture OR coach* OR certifi* OR "health promotion"):ti,ab,kw    Concept #3 (combine with OR):  [mh "Analgesics, Opioid"] OR [mh "Opiate Alkaloids"] OR [mh "Opiate Overdose"] OR [mh "Naloxone"]  (opioid* OR opiate* OR heroin OR fentanyl OR oxycodone OR hydrocodone OR morphine OR codeine OR naloxone OR narcan OR narkan OR talwin):ti,ab,kw    Concept #4:   (#2 AND #3) OR (([mh "Naloxone"] OR (naloxone OR narcan OR narkan OR talwin):ti,ab,kw AND (distribution OR provision OR dispensing OR delivery):ti,ab,kw)    Final Search Strategy:   #1 AND #4 | 1268 trials on 01/06/2023 |
| ProQuest ERIC | Concept #1 (combine with OR):  MAINSUBJECT.EXACT("Computer Simulation") OR MAINSUBJECT.EXACT("Computer Assisted Instruction")  OR MAINSUBJECT.EXACT.EXPLODE("Distance Education") OR MAINSUBJECT.EXACT.EXPLODE("Nontraditional Education") OR MAINSUBJECT.EXACT.EXPLODE("Internet")  OR MAINSUBJECT.EXACT.EXPLODE("Social Media") OR (MAINSUBJECT.EXACT("Learning Management Systems") OR MAINSUBJECT.EXACT("Computer Software")) OR (MAINSUBJECT.EXACT.EXPLODE("Mobile Classrooms") OR MAINSUBJECT.EXACT.EXPLODE("Mobile Educational Services")) OR MAINSUBJECT.EXACT("Handheld Devices") OR MAINSUBJECT.EXACT.EXPLODE("Electronic Mail") OR (MAINSUBJECT.EXACT("Telecommunications") OR MAINSUBJECT.EXACT("Computer Mediated Communication") OR MAINSUBJECT.EXACT("Teleconferencing") OR MAINSUBJECT.EXACT("Technology") OR MAINSUBJECT.EXACT.EXPLODE("Educational Technology"))   tiab(virtual OR remote OR online OR web* OR computer* OR video* OR smartphone OR “text messag*” OR telephone* OR “mobile device” OR phone* OR “electronic mail” OR e-mail OR “mobile app*” OR hotline* OR helpline* OR “call center*” OR e-health OR m-health OR “mobile health” OR telemedic* OR telenursing OR teleconferenc* OR telehealth OR “digital health” OR website OR software OR technolog* OR “augmented reality” OR internet OR “social media”) OR if(virtual OR remote OR online OR web* OR computer* OR video* OR smartphone OR “text messag*” OR telephone* OR “mobile device” OR phone* OR “electronic mail” OR e-mail OR “mobile app*” OR hotline* OR helpline* OR “call center*” OR e-health OR m-health OR “mobile health” OR telemedic* OR telenursing OR teleconferenc* OR telehealth OR “digital health” OR website OR software OR technolog* OR “augmented reality” OR internet OR “social media”)      Concept #2 (combine with OR):  MAINSUBJECT.EXACT("Professional Continuing Education") OR MAINSUBJECT.EXACT.EXPLODE("Patient Education") OR MAINSUBJECT.EXACT("Medical Education") OR MAINSUBJECT.EXACT("Professional Education") OR MAINSUBJECT.EXACT("Education") OR MAINSUBJECT.EXACT("Competency Based Education") OR MAINSUBJECT.EXACT("Academic Education") OR MAINSUBJECT.EXACT("Adult Education") OR MAINSUBJECT.EXACT("Comprehensive School Health Education") OR MAINSUBJECT.EXACT("Health Education") OR MAINSUBJECT.EXACT("Continuing Education") OR MAINSUBJECT.EXACT("Drug Education") OR MAINSUBJECT.EXACT("Inservice Education") OR MAINSUBJECT.EXACT("Community Education") OR MAINSUBJECT.EXACT("Courses") OR MAINSUBJECT.EXACT("Curriculum") OR MAINSUBJECT.EXACT("Training") OR MAINSUBJECT.EXACT("Professional Training") OR MAINSUBJECT.EXACT("Health Promotion") OR MAINSUBJECT.EXACT("Health Activities") OR MAINSUBJECT.EXACT.EXPLODE("Learning") OR MAINSUBJECT.EXACT.EXPLODE("Teaching Methods") OR MAINSUBJECT.EXACT.EXPLODE("Simulation")   tiab(educat* OR curricul* OR learn* OR training OR course OR teach* simulation OR instruc* OR “skill-building” OR “skills-building" OR lecture OR coach* OR certifi* OR “health promotion”) OR if(educat* OR curricul* OR learn* OR training OR course OR teach* simulation OR instruc* OR “skill-building” OR “skills-building" OR lecture OR coach* OR certifi* OR “health promotion”)    Concept #3 (combine with OR):  MAINSUBJECT.EXACT.EXPLODE("Narcotics")  tiab(opioid* OR opiate* OR heroin OR fentanyl OR oxycodone OR hydrocodone OR morphine OR codeine OR naloxone OR narcan OR narkan OR talwin) OR if(opioid* OR opiate* OR heroin OR fentanyl OR oxycodone OR hydrocodone OR morphine OR codeine OR naloxone OR narcan OR narkan OR talwin)    Concept #4:  (#2 AND #3) OR ((exp Naloxone/ OR tiab(naloxone OR narcan OR narkan OR talwin) AND tiab(distribution OR provision OR dispensing OR delivery))    Final Search Strategy: #1 (S3) AND #4 | 117 on 01/06/23 |
| CADTH Grey Matters | Opioid overdose response | 85 results, zero were relevant, on 27/07/23 |
| OpenGrey | Opioid overdose | 7 results, zero were relevant, on 27/07/23 |
| TRIP Pro | Online opioid overdose training | 327 results, 1 included (published after our search), on 27/07/23 |

## 
