# Supplementary material for: Virtual opioid poisoning education and naloxone distribution programs: A scoping review
Source: PLOS Digit Health. 2024 Jun 7;3(6):e0000412. doi: 10.1371/journal.pdig.0000412 (PMC11161022; doi:10.1371/journal.pdig.0000412)
Supplement: S3 Table — (DOCX) [file pdig.0000412.s003.docx]

## S3 Table. Data extraction instrument.

| Variables collected |
| --- |
| Title |
| Country |
| Objective |
| Study Design |
| Participants per group |
| Total number of participants |
| If there is a control group, what is the intervention received by this group? |
| Mode of delivery (e.g. individual, in groups) |
| Participants age |
| Participants age and/or gender |
| Participants ethnicity |
| Participants socioeconomic status |
| Participants area (e.g. rural, urban) |
| Type of participants (e.g. PWLLE, health professionals) |
| Intervention components (e.g. education only, naloxone distribution) |
| Intervention educational components |
| Intervention approach (e.g. video, website, mail) |
| Intervention duration |
| Reported outcomes |
| Scales used to report outcomes |
| Timepoint of outcome assessment |
| Qualitative summary of key findings |
| Study limitations and biases identified by authors |
| Study strengths |
